# Thyrotoxic periodic paralysis in a Caucasian man without identifiable genetic predisposition: a case report

**DOI:** 10.1186/s13044-023-00152-w

**Published:** 2023-05-01

**Authors:** Arne Heydorn, Birgitte Bertelsen, Rúna Louise Mortansdóttir Nolsöe, Pia Eiken, Peter Lommer Kristensen

**Affiliations:** 1 Department of Endocrinology and Nephrology, Nordsjællands Hospital, Dyrehavevej 29, DK-3400 Hillerød, Denmark; 2 Center for Genomic Medicine, Copenhagen University Hospital, Rigshospitalet, Copenhagen, Denmark; 3 Department of Endocrinology, Bispebjerg and Frederiksberg Hospital, Copenhagen, Denmark; 4 Department of Clinical Medicine, University of Copenhagen, Copenhagen, Denmark

**Keywords:** Thyrotoxic periodic paralysis, Hypokalemia, Graves’ disease, Muscle weakness, Periodic episodes, Hyperthyroidism, Channelopathies

## Abstract

**Background:**

Thyrotoxic periodic paralysis (TPP) is a rare condition characterized by muscle paralysis, thyrotoxicosis, and hypokalemia. It presents with paralysis of both proximal and distal musculature in upper and lower limbs and may affect respiratory musculature and the cardiac conduction system. Early diagnosis is essential, as the condition is potentially reversible by oral or intravenous potassium treatment, leading to rapid resolution without lasting weakness. Overlooking the diagnosis may result in respiratory failure and cardiac arrhythmias including QT prolongation, Torsades de points, and ventricular arrhythmias.

**Case presentation:**

A 19-year-old Caucasian man was admitted acutely with paralysis in upper and lower limbs and tachycardia. Over several months, he had experienced anxiousness, sweating more than usual, had daily palpitations, shortness of breath on exertion, and loose stools, and had lost 21 kg over the last year. Initial blood gas showed very low potassium of 1.4 mM, and blood tests showed decreased Thyroid-stimulating hormone (TSH) < 0.01 × 10^− 3^ IU/L, elevated free thyroxine (fT4) of 63.5 pM (reference interval (RI): 12.0–22.0 pM), and elevated total triiodothyronine (T3) of 8.2 nM (RI: 1.0–2.6 nM). He was diagnosed with TPP and treated with liquid oral potassium chloride (30 mmol every 30 minutes) and propylthiouracil (initial dose of 400 mg followed by 200 mg three times daily). TSH-receptor antibodies (TRAB) and thyroid-peroxidase antibodies (TPO-ab) were highly elevated. Thyroid ultrasound showed a normal-sized gland and color Doppler sonography showed increased vascularity throughout the gland, compatible with Graves’ disease. He was discharged on day 4 with a normal potassium level and followed in the outpatient clinic where he received standard care for Graves’ disease. Genetic testing using whole-genome sequencing found no genetic variants in genes previously associated with TPP.

**Conclusion:**

TPP is very rare in Caucasians but more often affects young men in East Asian populations. The case presents a Caucasian man with TPP where genetic testing of *CACNA1S*, *KCNJ18*, *SCN4A*, *KCNJ2*, *KCNE3*, and *ABCC8* shows no pathogenic variants in genes previously associated with TPP.

## Introduction

Thyrotoxic periodic paralysis (TPP) is a rare condition characterized by muscle paralysis, thyrotoxicosis, and hypokalemia. It presents with paralysis of both proximal and distal musculature in upper and lower limbs and may affect respiratory musculature and the cardiac conduction system. The condition is potentially reversible by oral or intravenous potassium treatment, leading to rapid resolution without lasting weakness. Overlooking the diagnosis may result in respiratory failure and cardiac arrhythmias including QT prolongation, Torsades de points, and ventricular arrhythmias. Most often the aetiology of thyrotoxicosis is Graves’ disease and there is a strong male preponderance, even though most people with Graves’ disease are women. High thyroid and beta-adrenergic activity can stimulate Na-K adenosine triphosphatase (ATPase) activity because thyroid hormones increase the number and sensitivity of beta receptors, which leads to increased catecholamine-mediated potassium uptake. This may explain why stress may precipitate episodes of TPP. Carbohydrate intake leading to hyperinsulinemia (which leads to muscle uptake of potassium) and an influx of potassium after exercise-related potassium release from muscles have been reported as precipitating episodes of TPP. Because the trans-membrane transport of potassium is changed in TPP, researchers have looked for genetically determined channelopathies involved in potassium transport in TPP patients. Until now several have been found [12].

### Case presentation

Here, we present a case of a 19-year-old Caucasian man who was admitted acutely around 5 am with paralysis in upper and lower limbs, with lower limbs most severely affected, and no other comorbidities. He had woken up in the night with spasms of the lower limbs, unable to get out of bed and unable to stand. He had experienced two similar episodes within the last year with remission of symptoms the following morning. He did not consult his general practitioner, thinking he had just slept on his arms and legs.

During the last year, he had lost 21 kg, from a weight of 82 kg (body mass index (BMI) 27.7 kg/m^2^) to 61 kg (BMI 20.6 kg/m^2^), a reduction of 26%. He had been intermittently anxious, sweating more than usual, had daily palpitations, shortness of breath on exertion, and loose stools. He had irritable/dry eyes and slight irritation when moving his eyes. There was no prior steroid intake, no heavy alcohol use, and no history of diabetes or hypertension. The history revealed no prior factors or events to happen before the admission. His family history was unremarkable. None of his five siblings or parents had experienced similar symptoms and none had a history of thyroid illness, hypokalemia, or hypertension.

On examination in the emergency department, he was unable to stand. Muscle strength was reduced in the upper (Medical Research Council scale for muscle strength (MRC) 3/5) and lower limbs (MRC 2/5), and he was just able to lift his arms. He had no respiratory distress. Deep tendon reflexes were reduced in upper limbs and absent in lower limbs. Cranial nerve examination was normal, and there was no sensory loss. The thyroid gland was not enlarged or tender. Eyes were normal with no redness, swelling, or exophthalmos.

**Fig. 1:**
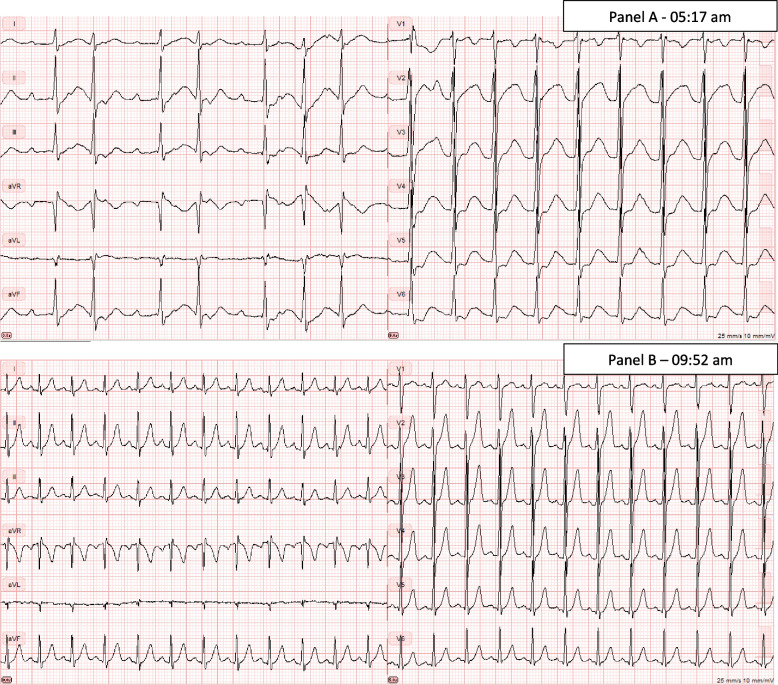
ECG of a 19-year-old Caucasian man with thyrotoxic periodic paralysis. Panel **A** shows the ECG at arrival to the Emergency Department with a potassium of 1.4 mM. Panel **B** (same day) shows the ECG after treatment with liquid oral potassium chloride (30 mmol every 30 minutes)

On admission, his vital signs were the following: Heart rate 120 bpm, blood pressure 104/45 mmHg, respiratory rate 16/min., temperature 36.6 °C, and oxygen saturation 99%. Electrocardiography (ECG) showed atrial flutter with varying 1:2 and 1:3 block (Fig. 1, panel A).

Initial blood gas showed potassium of 1.4 mM and slight metabolic acidosis with pH 7.34 and bicarbonate of 21 mM but was otherwise unremarkable. The initial venous lab values (Table 1) confirmed severe hypokalemia with potassium of 1.4 mM, normal sodium of 139 mM, normal magnesium, leukocytes and neutrophils elevated at 18.4 × 10^9^/L and 16.0 × 10^9^/L respectively, but with normal C-reactive protein (CRP) < 3 mg/L. Thyroid-stimulating hormone (TSH) was < 0.01 × 10^− 3^ IU/L, free thyroxine (fT4) and total triiodothyronine (T3) were elevated at 63.5 pM (reference interval (RI): 12.0–22.0 pM) and 8.2 nM (RI: 1.0–2.6 nM), respectively. TSH-receptor antibodies (TRAB) and thyroid-peroxidase antibodies (TPO-ab) were highly elevated. Alkaline phosphatase was elevated.

**Table 1:** Laboratory panel at admission of a 19-year-old Caucasian man with thyrotoxic periodic paralysis

Analyte (blood)	Patient result	↓↑	Reference interval
Leukocytes	18.4 × 10^9^/L	**↑**	3.5–8.8 × 10^9^/L
Neutrophils	16.0 × 10^9^/L	**↑**	1.6–5.9 × 10^9^/L
Sodium	139 mM		137–144 mM
Potassium	1.4 mM	**↓**	3.5–4.4 mM
Creatinine	50 μM	**↓**	60–105 μM
Urea	5.5 mM		3.2–8.1 mM
eGFR	> 90 mL/min		> 60 mL/min
Ionised calcium	1.26 mM		1.18–1.32 mM
Phosphate	1.53 mM		0.71–1.53 mM
Magnesium	0.71 mM		0.71–0.94 mM
Albumin	35 g/L	**↓**	36–48 g/L
Alanine aminotransferase	39 U/L		10–70 U/L
Alkaline phosphatase	167 U/L	**↑**	35–105 U/L
Lactate dehydrogenase	182 U/L		105–205 U/L
Amylase	59 U/L		25–120 U/L
Bilirubin	6 μM		5–25 μM
C-reactive protein	< 3 mg/L		< 10 mg/L
Glucose	7.8 mM	**↑**	4.2–7.2 mM
HbA1c	30 mmol/mol		< 48 mmol/mol
TSH	< 0.01 × 10^− 3^ IU/L	**↓**	0.4–4.8 × 10^− 3^ IU/L
Free T4	63.5 pM	**↑**	12.0–22.0 pM
Total T3	8.2 nM	**↑**	1.0 - 2.6 nM
TSH-receptor antibodies	> 100 IU/L	**↑**	< 0.7 IU/L
Thyroid-peroxidase antibodies	9.320 U/L	**↑**	< 60 U/L

Treatment was initiated in the emergency room with 30 mmol liquid oral potassium chloride. He was transferred to the Intensive Care Unit for ECG monitoring and treated with oral propranolol 10 mg every 2 hours and 30 mmol liquid oral potassium chloride every 30 minutes. Two hours after admission, potassium was normalized, potassium supplements were stopped, and he had regained power in both upper and lower limbs. The ECG changed to sinus rhythm with a frequency of 100–120 bpm (Fig. 1, panel B). He developed rebound hyperkalemia with a peak value of 6.9 mM 4 h after admission which normalized without treatment over 8 h. Propylthiouracil was started with an initial dose of 400 mg followed by 200 mg three times daily. Intravenous hydrocortisone was initially given at 100 mg every 8 h until a small suspicion of adrenal insufficiency based on a low systolic blood pressure and potentially precipitated by hyperthyroidism could be ruled out. Hydrocortisone was stopped the next day. He was discharged on day 4 with a normal potassium level and continued propylthiouracil 200 mg three times daily and propranolol 10 mg four times daily. Thyroid ultrasound showed a normally sized gland and color Doppler sonography showed increased vascularity throughout the gland, compatible with Graves’ disease. He was followed in the outpatient clinic, changed from propylthiouracil to thiamazole, and received standard care for Graves’ disease. Since TPP was suspected genetic testing was performed using whole genome sequencing (WGS) of DNA isolated from peripheral blood. An in silico panel of genes previously associated with thyrotoxic periodic paralysis was applied, including *CACNA1S* (NM_000069.3), *KCNJ18* (NM_001194958.2), *SCN4A* (NM_000334.4), *KCNJ2* (NM_000891.3), *KCNE3* (NM_005472.4), and *ABCC8* (NM_000352.6). Coding regions (+/− 20 bp of adjacent intronic regions) were analyzed for non-polymorphic single nucleotide variants as well as copy number variants. No genetic variants were identified within these regions and thus a genetic cause of the symptoms could not be established. Additionally, WGS showed as expected that the patient was of European descent.

## Discussion and conclusions

The aetiologies of hypokalemic periodic paralysis (HPP) can be divided into thyrotoxic periodic paralysis (TPP), hypernatremic hypokalemic paralysis (HHP), familial periodic paralysis (FPP) and idiopathic/sporadic periodic paralysis (SPP). Patients with SPP do not have hyperthyroidism, hypernatremia, or a family history of HPP. Among Caucasians, most cases of HPP are due to FPP, whereas in an Asian population most cases of HPP are due to TPP [3]. TPP is very rare in Caucasians.

We investigated the genetic predispositions for TPP previously documented in the literature, encompassing six different genes. Pathogenic variants in the *SCN4A* gene (sodium voltage-gated channel alpha subunit 4) and the *CACNA1S* gene (calcium voltage-gated channel subunit alpha1 S) can cause HPP. Pathogenic variants in *CACNA1S* cause up to 70%, and *SCN4A* pathogenic variants about 10% of all cases of HPP. HPP caused by *SCN4A* or *CACNA1S* variant are inherited in an autosomal dominant pattern [6].

The *KCNJ18* gene (potassium inwardly rectifying channel subfamily J member 18) encodes the inwardly rectifying potassium channel, Kir2.6. The locus is regulated by thyroid hormone, and variants in *KCNJ18* have been associated with TPP [4, 7, 10, 15]. A major haplotype of *KCNJ18* in East Asian populations is significantly associated with susceptibility to TPP, and this haplotype is much more common in East Asian than Caucasian populations [10].

The *KCNJ2* gene (potassium inwardly rectifying channel subfamily J Member 2) encodes the inwardly rectifying potassium channel, Kir2.1. Variants in the *KCNJ2* gene have been found to be significantly associated with TPP [11]. In a single study, a missense variant in the *KCNE3* gene (potassium voltage-gated channel subfamily E regulatory subunit 3) has been found to be associated with TPP [5]. Finally, a single study from 2014 found that a genetic variant of the *ABCC8* gene (subunit of the beta-cell ATP-sensitive potassium channel) which controls insulin secretion after feeding, was associated with susceptibility to TPP [14].

Using WGS followed by in silico panel analysis of coding regions we found no pathogenic variants in any of the above-mentioned genes in our patient. Hence, we did not find a genetic predisposition to TPP in this Caucasian man. The absence of a genetic predisposition in our patient is further supported by the fact that none of his five siblings or parents had ever experienced similar symptoms and none had a history of thyroid illness, hypokalemia, or hypertension.

TPP in Caucasians, although very rare, have been documented in earlier case reports, most recently in [1, 2, 8, 9, 13, 16]. Only in one case report by Rasheed et al. [13], a 27-year-old Irish man with TPP showed no genetic predisposition in *CACNA1S*, *SCN4A* and *KCNJ18*. Genetic analyses of *KCNJ2*, *KCNE3* and *ABCC8* was not done.

In the present case, treatment of hypokalaemia was done with liquid oral potassium chloride, 30 mmol every 30 minutes, which in a few hours led to rebound hyperkalaemia. In retrospect, the dose of potassium was too high. In some treatment protocols, the dose is advised to be at 30 mmol every 2 h with a maximum of 90 mmol in the first 24 hours of treatment.

In conclusion, we report the case of a young Caucasian man with TPP, without pathogenic variants in six genes previously reported to predispose to TPP, and without any relevant family history. Hence, we found no genetic explanation for why our patient developed TPP, suggesting that acquired environmental triggers or yet unidentified gene variants may play a role as co-factors in TPP pathogenesis. Having performed WGS we are able to reanalyze the sequencing data in the future, as new knowledge of predisposing genes appears. This may further the understanding of the genetic background of the rare occurrence of TPP in Caucasians.

## Data Availability

All data generated or analyzed during this study are included in the article.

## Abbreviations

CRP: C-reactive protein
RI: Reference Interval
T3: triiodothyronine
FT4: free thyroxine
TPO-ab: Thyroid-peroxidase antibodies
TPP: Thyrotoxic periodic paralysis (TPP)
TSH: Thyroid-stimulating hormone
TRAB: TSH-receptor antibodies
WGS: Whole genome sequencing
